# The Antibacterial Assay of Tectorigenin with Detergents or ATPase Inhibitors against Methicillin-Resistant *Staphylococcus aureus*

**DOI:** 10.1155/2014/716509

**Published:** 2014-05-28

**Authors:** Dae-Ki Joung, Su-Hyun Mun, Kuang-Shim Lee, Ok-Hwa Kang, Jang-Gi Choi, Sung-Bae Kim, Ryong Gong, Myong-Soo Chong, Youn-Chul Kim, Dong-Sung Lee, Dong-Won Shin, Dong-Yeul Kwon

**Affiliations:** ^1^Department of Oriental Pharmacy, College of Pharmacy and Wonkwang-Oriental Medicines Research Institute, Wonkwang University, Iksan, Jeonbuk 570-749, Republic of Korea; ^2^BK21 Plus Team, Professional Graduate School of Oriental Medicine, Wonkwang University, Iksan, Jeonbuk 570-749, Republic of Korea; ^3^Department of Third Medicine, Professional Graduate School of Oriental Medicine, Wonkwang University, Iksan, Jeonbuk 570-749, Republic of Korea; ^4^Standardized Material Bank for New Botanical Drugs, College of Pharmacy, Wonkwang University, Iksan, Jeonbuk 570-749, Republic of Korea; ^5^Department of Oriental Medicine Resources, Sunchon National University, Jeonnam 540-742, Republic of Korea

## Abstract

Tectorigenin (TTR) is an O-methylated isoflavone derived from the rhizome of *Belamacanda chinensis* (L.) DC. It is known to perform a wide spectrum of biological activities such as antioxidant, anti-inflammatory, anti-tumor. The aim of this study is to examine the mechanism of antibacterial activity of TTR against methicillin-resistant *Staphylococcus aureus* (MRSA). The anti-MRSA activity of TTR was analyzed in combination assays with detergent, ATPase inhibitors, and peptidoglycan (PGN) derived from *S. aureus*. Transmission electron microscopy (TEM) was used to monitor survival characteristics and changes in *S. aureus* morphology. The MIC values of TTR against all the tested strains were 125 **μ**g/mL. The OD(600) of each suspension treated with a combination of Triton X-100, DCCD, and NaN_3_ with TTR (1/10 × MIC) had been reduced from 68% to 80%, compared to the TTR alone. At a concentration of 125 **μ**g/mL, PGN blocked antibacterial activity of TTR. This study indicates that anti-MRSA action of TTR is closely related to cytoplasmic membrane permeability and ABC transporter, and PGN at 125 **μ**g/mL directly bind to and inhibit TTR at 62.5 **μ**g/mL. These results can be important indication in study on antimicrobial activity mechanism against multidrug resistant strains.

## 1. Introduction

Methicillin-resistant* Staphylococcus aureus *(MRSA) can lead to considerable morbidity and mortality in orthopaedic patients. The mortality rate from MRSA bacteraemia is double that of methicillin-sensitive* Staphylococcus aureus* (MSSA) [1]. The complication rate and cost of periprosthetic joint infection with MRSA is considerably higher compared to those of MSSA [2]. Patients receiving orthopaedic implants are most vulnerable, given the potential for biofilm formation and long-term morbidity [3, 4]. Yet, the incidence of MRSA in orthopaedic patients has increased [5]. MRSA strains have become resistant not only to *β*-lactam antibiotics but also to fluoroquinolones and other families of antibiotics [6].

Tectorigenin (TTR), an* O*-methylated isoflavone, has been shown to possess antioxidant, anti-inflammatory, antitumor activities and has selective estrogen receptor modulator activities [7–10]. TTR significantly decreased in the concentration of DPPH radical on the scavenging ability of the tectorigenin, inhibited the IFN-*γ*/LPS-induced NO, COX-2, PGE2, and IL-1*β* production in the activated macrophages RAW 264.7 cells, and caused a significant inhibition of tumor growth in LLC bearing C57BL/6 mice [11]. It showed the inhibitory effect of isoflavones isolated from the* Pueraria thunbergiana* (Leguminosae) against* Helicobacter pylori *[12]. In this study, we used TTR that isolated from rhizome of* Belamcanda chinensis*, it was called drug for eliminating sputum as well as clearing away heat and detoxicating [13]. TTR can be isolated from* Pueraria thunbergiana*,* Belamcanda chinensis*, or* Pueraria lobata* [14–16]. Although it has many biological activities, TTR has not yet been reported to have antibacterial activity on MRSA, multidrug-resistant pathogen.

In the present study, to clarify the mechanism of anti-MRSA activity of TTR, we investigated the antibacterial activities of TTR on the membrane-binding agent and ATPase-inhibiting agents. In addition, we also investigated the effects of adding peptidoglycan (PGN) derived from* S. aureus* into Mueller-Hinton broth (MHB) that contained TTR alone. In this study, we aimed to gain insights into the antibacterial activity, survival characteristics, and changes in bacterial morphology and mechanism of TTR against MRSA.

## 2. Materials and Methods

### 2.1. Isolation and Purification of Tectorigenin

**Figure 1:**
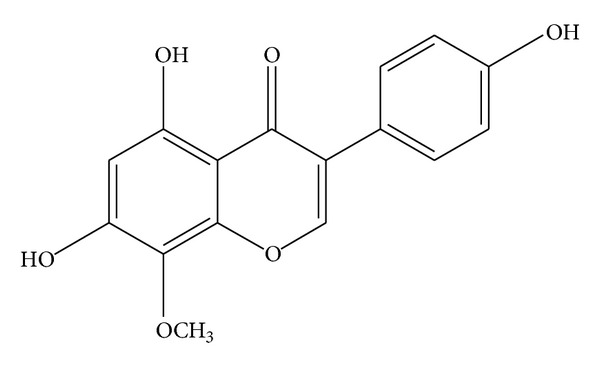
Chemical structure of tectorigenin.

Tectorigenin (>95%) was deposited at the Standardized Material Bank for New Botanical Drugs (number NNMBP000017) at Wonkwang University (Iksan, Republic of Korea). The dried rhizome of* Belamcanda chinensis* was purchased from the University Oriental Drugstore, Iksan, Korea, in June 2006 and was positively identified by Professor Youn-Chul Kim, College of Pharmacy, Wonkwang University. A voucher specimen (number WP06-189) was deposited at the Herbarium of the College of Pharmacy, Wonkwang University (Korea). The dried rhizome of* B. chinensis* (1 kg) was extracted twice with 70% aqueous EtOH (2 L) under the ultrasonic condition for 3 h. The 70% EtOH extract (259 g) was suspended in H_2_O (1 L) and partitioned successively with *n*-hexane (800 mL × 2) and CHCl_3_ (800 mL × 2) to yield *n*-hexane soluble (7.71 g) and CHCl_3_-soluble extract (41.94 g). The CHCl_3_-soluble extract was subjected to column chromatography (CC) on silica gel, which was using *n*-hexane-EtOAc (4 : 1→2 : 1) to give four fractions (Fr. A–D). Fr. A (1.75 g) was subjected to silica gel CC with CHCl_3_-MeOH (25 : 1) to get three subfractions (Fr. A1–A3). Fr. A2 (540 mg) was further chromatographed on a Sephadex LH-20 with CHCl_3_-MeOH (25 : 1) to afford tectorigenin (387 mg, 0.0387 w/w%). The structure of tectorigenin was identified by comparison of its ^1^H- and ^13^C-NMR data with those reported in the literature [17]. The purity (99.46%) of tectorigenin was determined by HPLC (Figure 1). 

*Tectorigenin.* Pale yellow solid, (−)-ESI-MS* m/z* 299 [M-H]^−^, ^1^H-NMR (DMSO-*d*_6_, 500 MHz) *δ*: 8.32 (1H, s, H-2), 7.37 (2H, d, *J* = 8.7 Hz, H-2′, 6′), 6.82 (2H, d, *J* = 8.7 Hz, H-3′, 5′), 6.49 (1H, s, H-8), 3.74 (3H, s, OCH_3_); ^13^C-NMR (DMSO-*d*_6_, 125 MHz) *δ* 181.1 (C-4), 158.0 (C-9), 157.9 (C-4′), 154.6 (C-2), 153.8 (C-7), 153.3 (C-5), 131.9 (C-6), 130.7 (C-2′, 6′), 122.3 (C-3), 121.7 (C-1′), 115.6 (C-3′, 5′), 105.4 (C-10), 94.4 (C-8), and 60.5 (OCH_3_).

### 2.2. Reagent

Ampicillin (AM), gentamicin (GT), ciprofloxacin (CP), Triton X-100 (TX-100),* N*,*N*′-dicyclohexylcarbodiimide (DCCD), and lipopolysaccharide (LPS) were purchased from Sigma-Aldrich Co. (St. Louis, USA). Mueller-Hinton broth (MHB) was purchased from Difco (Baltimore, MD, USA). Tris(hydroxymethyl) aminomethane (TRIS) was obtained from AMRESCO (San Francisco, CA). Sodium azide (NaN_3_) and PGN were purchased from Fluka (Switzerland).

### 2.3. Bacterial Strains and Growth Conditions

Three clinical isolates of MRSA were obtained from three different patients at Wonkwang University Hospital (Iksan, South Korea). The other two strains were* S. aureus* ATCC 33591 (methicillin-resistant strain) and* S. aureus* ATCC 25923 (methicillin-susceptible strain). Before use, all bacteria were stored in 30% glycerol and frozen at −70°C and were cultured in Mueller-Hinton broth (MHB) and Mueller-Hinton agar (MHA) (Difco Laboratories, Baltimore, MD, USA) and incubated at 37°C for 24 h for each experiment.

### 2.4. Minimum Inhibitory Concentration

The minimum inhibitory concentration (MIC) was determined using the broth microdilution method according to the Clinical and Laboratory Standards Institute guideline (CLSI, 2006) [18]. TTR was diluted by MHB in 96-well plate (0.5% [w/v] stock concentration). Preparation of the microorganism suspension was prepared by growing microorganism in broth for 24 h, and the suspensions were adjusted to a 0.5 McFarland standard turbidity (approximately 1.5 × 10^8^ CFU/mL). Final inoculums were adjusted to the 1.5 × 10^6^ CFU/mL. The plates were then incubated along with inoculum at 37°C for 18 h. MIC was defined at the lowest concentration of antibiotics and TTR. At the end of each incubation period, the well plates were visually examined for turbidity. Cloudiness indicated that bacterial growth has not been inhibited by the concentration of antimicrobial agent in the medium.

### 2.5. Antibacterial Activity with Detergent or ATPase Inhibitors

To elucidate whether the antibacterial activity of TTR was associated with either the altered membrane permeability or the mechanism of multidrug resistance (MDR), we examined the antibacterial activity of TTR in the presence of detergents and ATPase-inhibiting agents, respectively. To determine the detergent-induced permeabilization, a particular concentration of TTR was determined using the detergent TX-100 [19]. The nonionic detergent TX-100 greatly increases antibiotic sensitivity [20]. DCCD and NaN_3_, a metabolic inhibitor that can decrease ATP levels by disrupting electrochemical proton gradients in a bacterial environment, were used as inhibitors of ATPase [21, 22]. The antibacterial activity of TTR was measured in the presence of 0.01% TX-100, 0.0015% NaN_3_, and 6.25 *μ*g/mL DCCD.

### 2.6. Effect of Exogenous Peptidoglycan on TTR Activity

To determine the activity of exogenous PGN in the presence of TTR, TTR+PGN combination assays were performed using the method by Zhao et al. [23]. This assay indicated whether TTR directly binds PGN and interrupted integrity of cell wall. TTR was added to PGN by serial dilution. LPS was used as a control.

### 2.7. Transmission Electron Microscopy (TEM)

MRSA exponential-phase cultures were prepared by diluting cultures into MHB overnight, which was continued at 37°C until the cultures reached the midlogarithmic phase of growth. MHB-grown exponential-phase MRSA was treated with 1/2 × MIC and 1 × MIC of TTR for 30 min. Following the treatment, 2 mL of the culture was collected by centrifugation at 10,000 g for 10 min. After removal of the supernatant, pellets were fixed with modified Ki Woo Kim fixative [24]. Specimens were examined with an energy-filtering transmission electron microscope (LIBRA 120; Carl Zeiss, Oberkochen, Germany) at an accelerating voltage of 120 kV. Transmitted electron signals were recorded using a 4 k × 4 k slow-scan charge-coupled device camera (Ultrascan 4000 SP; Gatan, Pleasanton, CA) attached to the electron microscope.

## 3. Results

### 3.1. The MICs of TTR

The MIC values of TTR against all strains were 125 *μ*g/mL. All strains were resistant to AM, GT, and CP with MIC ranging from 31.25 to 1,000 *μ*g/mL (Table 1).

### 3.2. Antibacterial Activity with Detergent or ATPase Inhibitors

**Figure 2:**
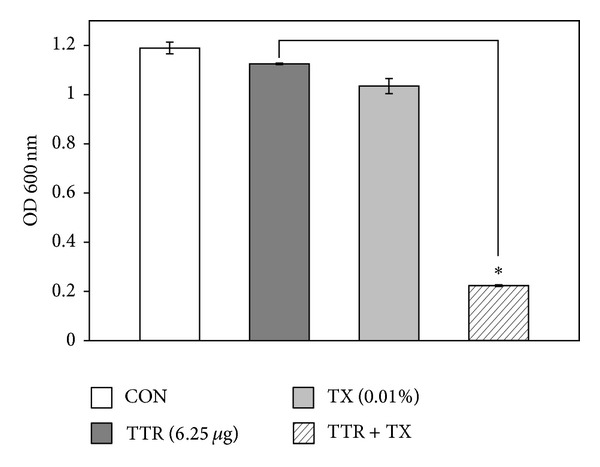
The effect of the membrane-permeabilizing agent Triton X-100 (TX-100) on the susceptibility of* Staphylococcus aureus* (ATCC 33591) to tectorigenin (TTR) treatment. The viability of bacteria was determined spectrophotometrically (optical density at 600 nm, OD_600_) after incubation for 36 h with 6.25 *μ*g/mL TTR and 0.01% Triton X-100. The data is represented as an average of three independent experiments. ^∗^ *P* < 0.001 as compared to TTR alone.

**Figure 3:**
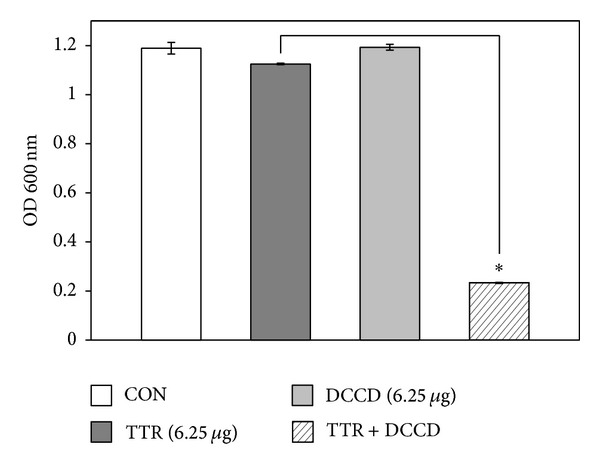
The effect of the ATPase-inhibitor* N,N*′-dicyclohexylcarbodiimide (DCCD) on the susceptibility of* Staphylococcus aureus* (ATCC 33591) to tectorigenin (TTR) treatment. The viability of bacteria was determined spectrophotometrically (optical density at 600 nm, OD_600_) after incubation for 36 h with 6.25 *μ*g/mL TTR and 6.25 *μ*g/mL DCCD. The data is represented as an average of three independent experiments. ^∗^ *P* < 0.001 as compared to TTR alone.

**Figure 4:**
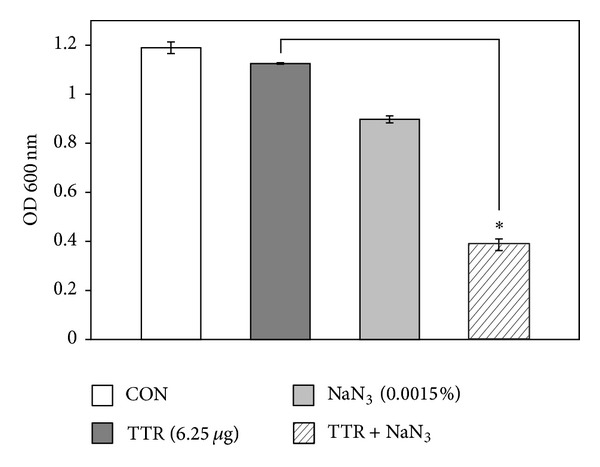
Effect of the ATPase-inhibitor NaN_3_ on the susceptibility of* Staphylococcus aureus* (ATCC 33591) to tectorigenin (TTR). The viability of bacteria was determined spectrophotometrically (optical density at 600 nm, OD_600_) after incubation for 36 h with 12.5 *μ*g/mL TTR and 0.0015% NaN_3_. The data is average of three independent experiments. ^∗^ *P* < 0.001 as compared to TTR alone.

The increased effect of detergent-induced membrane permeability on the activity of TTR is shown in Figure 2. We noted a modest reduction in the OD_600_ value of TTR-treated suspensions. Compared to the OD_600_ value of TTR alone (6.25 *μ*g/mL), the OD_600_ value of suspensions treated with the combination of TX-100 and TTR was reduced to 80%. Bacterial viability in the presence of TTR with DCCD and NaN_3_ as metabolic inhibitors had been reduced to 79% and 68%, respectively, compared to that with TTR alone (Figures 3 and 4).

### 3.3. The Binding of TTR and Peptidoglycan (PGN) with TTR

**Figure 5:**
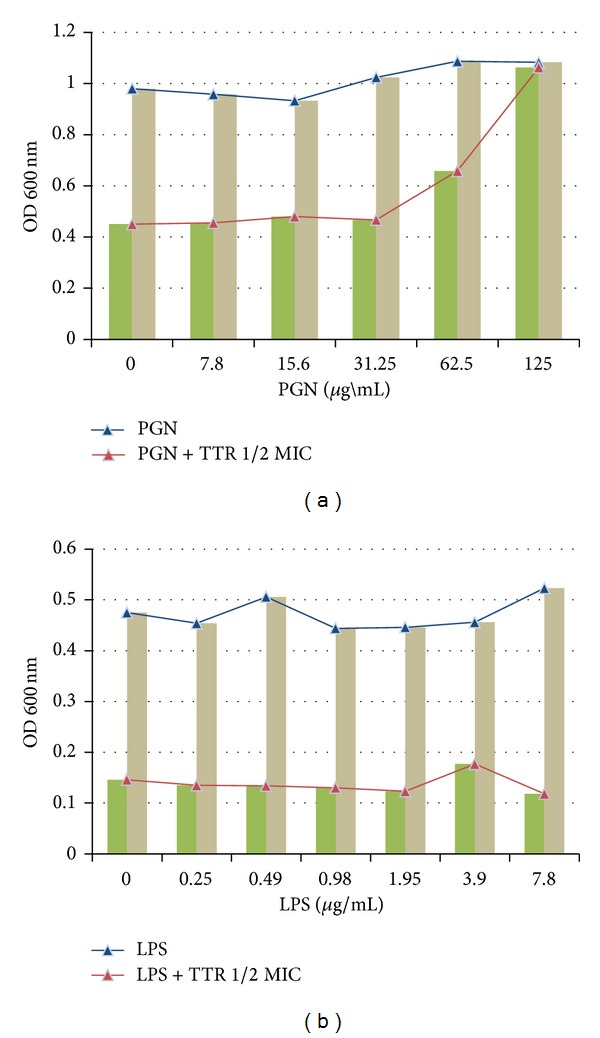
The binding effect of tectorigenin (TTR) with peptidoglycan (PGN) of the cell wall of* Staphylococcus aureus* (*S. aureus*). Lipopolysaccharide (LPS) was used as a control.

The binding of TTR to PGN was confirmed by adding PGN (0–125 *μ*g/mL) derived from* S. aureus* into MHB containing TTR alone (62.5 *μ*g/mL). At a concentration of 125 *μ*g/mL, PGN inhibited the antibacterial activity of TTR, whereas LPS did not show any inhibitory effects (Figure 5).

### 3.4. TEM of MRSA

**Figure 6:**
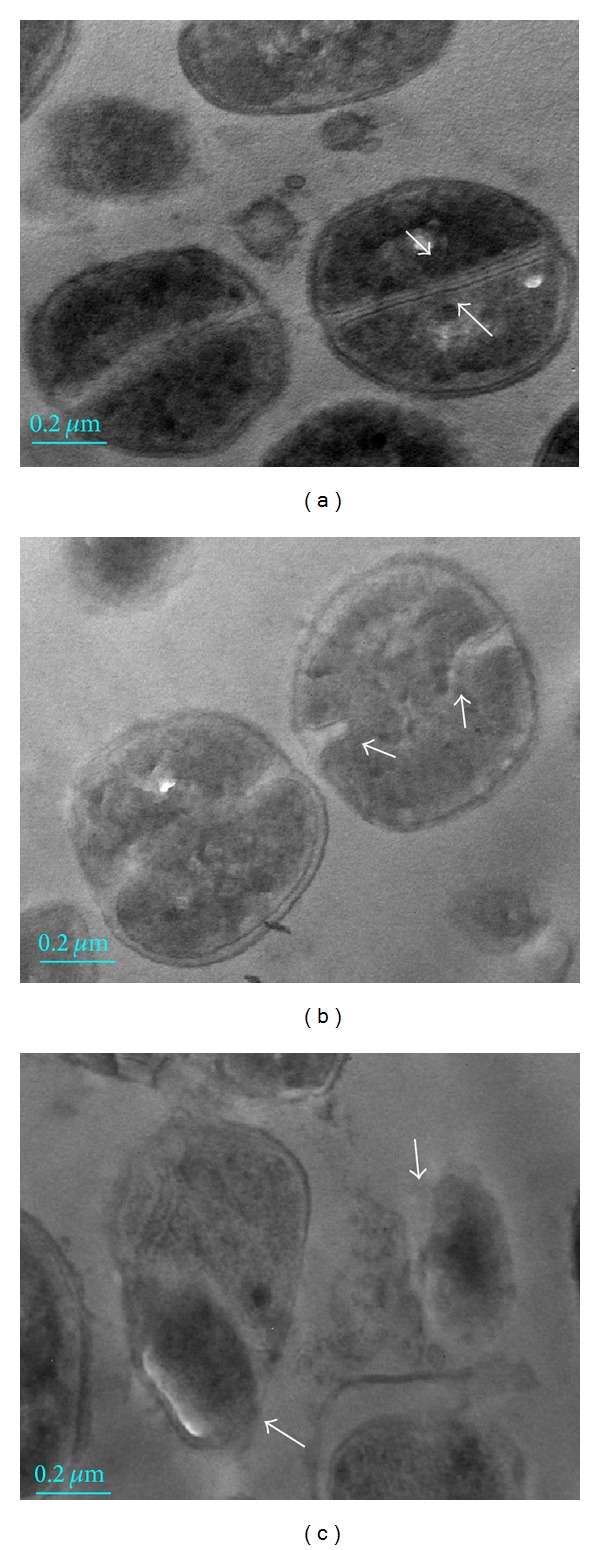
Transmission electron microscopy (TEM) images of methicillin-resistant* Staphylococcus aureus* (MRSA) (ATCC 33591) after 24 h of tectorigenin (TTR) treatment. (a) MRSA in the untreated control appeared to have intact membrane; (b) MRSA treatment with 1/2 MIC TTR (62.5 *μ*g/mL) hampered membrane integrity and caused membrane damage; (c) MRSA treatment with MIC TTR (125 *μ*g/mL) showed cytoplasmic membrane disruption and separated cell.

The control cells had normal morphology of* S. aureus* with distinct septa (Figure 6(a)). However, MRSA cells treated with TTR had decreased numbers of distorted septa (Figure 6(b)). Distinct septa formation was rarely observed in the treated cells. Ghost cells and decreased cell divisions were more frequently observed in TTR exposed cells (Figure 6(c)) compared to the control.

## 4. Discussion

Generally, antibacterial drugs inhibit bacterial growth in different targets, including inhibition of cell wall synthesis, disruption of cell membrane function, inhibition of protein, and nucleic acid synthesis [25]. According to our* in vitro* results, TTR showed the anti-MRSA action by increasing cytoplasmic membrane permeability and inhibiting adenosine triphosphatase (ATPase). MRSA (ATCC 33591) viability was highly decreased when TTR (6.25 *μ*g/mL) and detergent TX-100 were used together. The nonionic detergent TX-100 helps to extract membrane protein [26]. TX-100 selectively solubilizes cytoplasmic membrane proteins and increase membrane permeability from strains. It reduces methicillin resistance and stimulates autolysis [27]. The cellular autolysin activity of TX-100 potentiated susceptibility to TTR in growth of MRSA. Growth of MRSA (ATCC 33591) in 0.01% TX-100 alone had no effect on cell viability. This result suggests that TX-100 induces the release of autolysin inhibitor lipoteichoic acid (LTA) in the membrane bilayer. LTA is a major constituent of the gram-positive cell wall and is attached to the peptidoglycan layer. We also used* N,N*-dicyclohexylcarbodiimide (DCCD) and NaN_3_ as inhibitors of ATP synthase in the bacterial cells [21, 28]. DCCD disturbs H^+^-translocating sector (F_0_) of the F_0_F_1_-ATP synthase of coupling membranes. Most bacteria produce ABC (ATP-binding cassette) transporter that is an essential uptake system for amino acids in bacterial membrane [29]. These transporters cause the antibiotic resistant bacteria [30]. The antibacterial activity of TTR against MRSA in the presence of DCCD was markedly increased (Figure 3). The reason for increased susceptibility of MRSA toward TTR is the inhibition of ABC transporters that have ATP-dependent transporting activity by DCCD. NaN_3_ is a metabolic inhibitor, which reduces ATP level by disrupting electrochemical proton gradients in a bacterial cell [31, 32]. Actually, it has been reported that 0.001% NaN_3_ significantly increased susceptibility toward silybin in clinical* Pseudomonas aeruginosa* isolates [22].

Peptidoglycan (PGN) and lipoteichoic acid (LTA) are main components of gram-positive cell wall [33]. The cell wall of gram-positive bacteria including* S. aureus* consists of glycan layers, up to 30 sheets, and plays an essential role not only in osmotic protection but also in cell division. Cell envelope of* S. aureus* is surrounded by thick-layer of cross-linked peptidoglycan, whereas in gram-negative bacteria, the PGN layer is thin and is overlaid by an outer membrane composed mainly of lipopolysaccharide (LPS) [34–36]. As shown in Figure 5, TTR alone (62.5 *μ*g/mL) greatly inhibited more than 50% of the growth of* S. aureus*, but PGN (125 *μ*g/mL) from* S. aureus* blocked the antibacterial activity of TTR. These results indicate that direct binding with PGN and TTR completely blocked TTR-induced damage of the bacterial cell wall.

Transmission electron microscopy (TEM) can provide useful insight into the mechanism of action of antimicrobial agents. It is known that, when bacteria are exposed to antimicrobial agent at low concentrations, changes have been observed for bacterial morphology, ultrastructure, biochemistry, and multiplication rate [34]. Antibiotic treatment induces other cellular changes, such as cell lysis and separation of cytoplasmic contents from the membrane [25]. TEM images of MRSA confirmed that cytoplasmic membrane disruption and cell lysis of MRSA following exposure to the TTR. Thus, the mechanism of antibacterial activity of TTR involved the membrane disruption and cell lysis.

The overall results of the present study show that TTR has anti-MRSA activity. The results of TTR treatment in combination with TX-100 or DCCD and NaN_3_ showed that TTR has role in increasing cytoplasmic membrane permeability and decreasing activity of ABC transporter. These results show the promising effect for the use of TTR-based products in the treatment of MRSA. Further,* in vivo* experiments are needed for the clinical use of TTR on MRSA-infected patients.

## Figures and Tables

**Table 1:** MIC of *S. aureus* strains used in experiments.

*S. aureus* strains	Class	MIC (*μ*g/mL)			
		TTR	AM	GT	CP
ATCC 25923	MSSA	125	31.25	62.5	31.25
ATCC 33591	MRSA	125	1,000	31.25	500
DPS-1	MRSA	125	1,000	250	125
DPS-2	MRSA	125	1,000	125	125
DPS-3	MRSA	125	1,000	125	125

TTR: tectorigenin; AM: ampicillin; GT: gentamicin; CP: ciprofloxacin; DPS: *Staphylococcus aureus* strains from the Department of Plastic Surgery, Wonkwang University Hospital.
